# Phase-lags in large scale brain synchronization: Methodological considerations and in-silico analysis

**DOI:** 10.1371/journal.pcbi.1006160

**Published:** 2018-07-10

**Authors:** Spase Petkoski, J. Matias Palva, Viktor K. Jirsa

**Affiliations:** 1 Aix-Marseille Université, Inserm, INS UMR_S 1106, Marseille, France; 2 Neuroscience Center, Helsinki Institute of Life Science, University of Helsinki, Helsinki, Finland; Ghent University, BELGIUM

## Abstract

Architecture of phase relationships among neural oscillations is central for their functional significance but has remained theoretically poorly understood. We use phenomenological model of delay-coupled oscillators with increasing degree of topological complexity to identify underlying principles by which the spatio-temporal structure of the brain governs the phase lags between oscillatory activity at distant regions. Phase relations and their regions of stability are derived and numerically confirmed for two oscillators and for networks with randomly distributed or clustered bimodal delays, as a first approximation for the brain structural connectivity. Besides in-phase, clustered delays can induce anti-phase synchronization for certain frequencies, while the sign of the lags is determined by the natural frequencies and by the inhomogeneous network interactions. For in-phase synchronization faster oscillators always phase lead, while stronger connected nodes lag behind the weaker during frequency depression, which consistently arises for in-silico results. If nodes are in anti-phase regime, then a distance *π* is added to the in-phase trends. The statistics of the phases is calculated from the phase locking values (PLV), as in many empirical studies, and we scrutinize the method’s impact. The choice of surrogates do not affects the mean of the observed phase lags, but higher significance levels that are generated by some surrogates, cause decreased variance and might fail to detect the generally weaker coherence of the interhemispheric links. These links are also affected by the non-stationary and intermittent synchronization, which causes multimodal phase lags that can be misleading if averaged. Taken together, the results describe quantitatively the impact of the spatio-temporal connectivity of the brain to the synchronization patterns between brain regions, and to uncover mechanisms through which the spatio-temporal structure of the brain renders phases to be distributed around 0 and *π*.

**Trial registration:** South African Clinical Trials Register: http://www.sanctr.gov.za/SAClinicalbrnbspTrials/tabid/169/Default.aspx, then link to respiratory tract then link to tuberculosis, pulmonary; and TASK Applied Sciences Clinical Trials, AP-TB-201-16 (ALOPEXX): https://task.org.za/clinical-trials/.

## Author summary

Functional connectivity, and in particular, phase coupling between distant brain regions may be fundamental in regulating neuronal processing and communication. However, phase relationships between the nodes of the brain and how they are confined by its spatio-temporal structure, have been mostly overlooked. We use a model of oscillatory dynamics superimposed on the space-time structure defined by the connectome, and we analyze the possible regimes of synchronization. Limitations of data analysis are also considered and we show that the choice of the significance threshold for coherence does not essentially impact the statistics of the observed phase lags, although it is crucial for the right detection of statistically significant coherence. Analytical insights are obtained for networks with heterogeneous time-delays, based on the empirical data from the connectome, and these are confirmed by numerical simulations, which show in- or anti-phase synchronization depending on the frequency and the distribution of time-delays. Phase lags are shown to result from inhomogeneous network interactions, so that stronger connected nodes generally phase lag behind the weaker.

## Introduction

Many processes in nature are oscillatory, from heart beats and birds flapping their wings, to firing of neurons [1] and brain rhythms [2]. Oscillators are rarely isolated and they interact when coexisting in the same environment, thus synchronizing by adjusting their rhythms [3]. Synchronization, or consistent phase relationships, of distant regions of the brain has been detected by a variety of measures and may be a key mechanism for the regulation of cortical processing and communication [4, 5]. Advances of non-invasive structural brain imaging [6, 7] have made feasible large-scale network modeling approaches using biologically realistic connectivity, defined by the connection topology and delays, the so-called connectome, which is a crucial determinant of the network behavior [8–13]. The Kuramoto model (KM) [14] as a paradigm for the emergent group dynamics of coupled oscillatory subsystems [15, 16] is well suited for assessing how the connectome governs the brain oscillatory dynamics [17–22], which is then reflected in the phase relationship between brain regions.

Studies of networks dynamics predominantly focus on the synchronization properties, while the actual phase relationship between the oscillators is typically ignored, especially in complex networks with delays [16, 23]. For the tractable case of all-to-all equally coupled phase oscillators in thermodynamic limit, the phases of each oscillator are either constantly shifted from the mean phase, or non-uniformly rotate with a speed dependent on their natural frequencies, while still preserving the overall stationary distribution [14]. For heterogeneous couplings, phases become multimodal [24] and thus imply multimodal phase shifts for stationary synchronization, and for couplings of mixed signs [25], the oscillators generally split at distance *π*, but for strong coupling they form a traveling wave. Glassy states with ordered, but uniformly distributed phases for each frequency also appear for distributed parameters [26], and for structured networks multiple mean fields appear with oscillating, bounded or unbounded phase differences between them [27].

A number of computational studies on brain functional connectivity use neural masses connected with connectome-defined delays and weights. This yields intermittent in- or anti-phase synchronization [8] and a good agreement with experimental studies of phase relationship between local node dynamics and their degree in healthy subjects [28], and for Alzheimer’s disease [29]. Time-delays have been employed as a necessary condition for modeling anti-phase spatio-temporal patterns in the brain [11, 30], and for pair-wise coherence in connectome networks of phase oscillators that reproduce resting state patterns in BOLD fMRI [19], MEG [31] and EEG [21, 32, 33].

Oscillatory processes are particularly sensitive to delays, because shifts in phasing may render excitatory connections to inhibitory, and vice versa. The impact of time-delays on the synchronization, and indirectly to the phase-lags, has been studied for a single delay [34, 35] or for homogeneously distributed delays [36, 37], but so far only for all-to-all connectivities or for simple motifs. Spatially heterogeneous delays are particularly important for number of systems, foremost the brain [10, 11, 13]. Depending on their distribution, time-delays impose phase-shifted, in- or anti- phase clusters of oscillators, but their impact for phase-lags in large-scale neural synchronization has not been properly investigated. Stronger connected network nodes have been demonstrated to lag behind the weaker for randomly distributed delays shorter than a quarter period of the oscillators [22], but this restricts a large portion of the relevant frequencies.

In the current study we identify the relationship of the brain topology and its spatio-temporal structure, with the phase lags between the brain regions at any frequency of the brain processes. Analytical insights of synchronization on networks with distributed delays and heterogeneous couplings and frequencies, are applied to in-silico large-scale brain dynamics. Phase lockings and lags are studied in consideration to the limitations of time-series analysis that depend on the regime and levels of coherence. Inhomogeneous interactions due to the connectome are shown to drive the phase relationship, whilst the regimes of synchronization are constrained by the organization of the time-delays. Besides in-phase, these include anti-phase locking that for weak coherence depends on the length of the links, while for strong coupling is prevalent for the nodes of opposite hemispheres.

## Model

Spatio-temporal organization of delay-coupled oscillatory networks is often studied via phase oscillators that arise for weak interactions [14, 38–40]. The delays are reduced to phase shifts when they are small [38, 39, 41], but they appear inside the state variables when they are of the order of 1/coupling-strength [38, 39], yielding

$$\begin{array}{c} {\dot{\theta }}_{i}\left(t\right)=\omega_{i}+\epsilon h_{i}\left(\theta_{1}\left(t-\tau_{i,1}\right),\theta_{2}\left(t-\tau_{i,2}\right)\ldots \theta_{n}\left(t-\tau_{i,N}\right),K_{i,1},K_{i,2}\ldots K_{i,N}\right),\;i=1\ldots N, \end{array}$$ (1)

where *ω*_*i*_ are the natural frequencies, and for each link *K*_*ij*_ and *τ*_*ij*_ are coupling strengths and time-delays. Phase models still exhibit rich behavior and a direct link to more complex biophysical models, while admitting analytic approaches [40–43]. A special case is the Kuramoto model, which keeps only the first sine term of the Fourier series of *h*_*i*_ and that has been worked out to allow full analytical tractability. A large class of oscillators that are near an Andronov-Hopf bifurcation can be exactly transformed to the KM [14, 44], as well as Wilson–Cowan networks [41, 45]. Although the KM is not explicitly a brain model, it has been applied as one [18, 19, 21, 31–33] since it is perfectly suited to describe large-scale network synchronization. The utilized model therefore reads

$$\begin{array}{c} {\dot{\theta }}_{i}=\omega_{i}+\frac{1}{N}\sum_{j=1}^{N}K_{ij}\text{sin}\left[\theta_{j}\left(t-\tau_{ij}\right)-\theta_{i}\right],\;\;i=1\ldots N, \end{array}$$ (2)

where network interactions are symmetric with *K*_*ij*_ = *K*_*ji*_ and *τ*_*ij*_ = *τ*_*ji*_. The aim of the model is to show correspondence to the empirical results for phase difference. These are often captured by phase locking values [46], which are a statistical measure for similarity between phases of two signals. For 1: 1 synchronization, which is of main interest in the empirical studies, PLV is defined as

$$\begin{array}{c} cPLV_{ij}\equiv PLV_{ij}{\text{e}}^{i\phi_{ij}}=\frac{1}{M}\sum_{p=1}^{M}{\text{e}}^{i\Delta \theta_{ij}\left(p\right)}, \end{array}$$ (3)

where the phase difference Δ*θ*_*ij*_(*p*) = *θ*_*i*_(*p*) − *θ*_*j*_(*p*) is calculated at times *p* = 1…*M*.

### Numerical analysis and statistics

Numerical integrations utilize a Heun scheme adapted to time delays. The time-step is set as 0.01/(max([max(*K*), 0.05*μ*, *D*, 1])), with noise intensity *D* and mean of the natural frequencies *μ*, thus assuring that it is never larger than 0.01*s* and it is accordingly decreased for larger couplings, frequencies or noise. All time series are down-sampled to twice the Nyquist frequency of the fastest oscillator.

Complex phase locking values, Eq (3), are calculated at sliding windows of length equal to 10 periods of the mean entrainment frequency and with 75% overlap. Qualitatively similar results are obtained for windows lengths between 5 and 10 periods, and for overlapping between 50% and 90%, although longer windows yield systematically lower level for statistical significance [47].

Signals can often be coherent just by chance and statistical testings are necessary to correctly identify the coherence due to the mutual interactions [46, 47]. The level of significance for PLV is calculated as the 95th percentile of maximum values in 100 surrogate signals using two different procedures, followed by the same processing as for the original time-series. The first surrogates, which yield less strict level of significance are obtained by shuffling the time series of the phases of one of the oscillators. The second, generally stricter level is obtained by two independent uncoupled oscillators with the same frequencies, fixed or time-varying, as the original oscillators, with the same level of noise. The problem with the latter is that in empirical analysis the parameters of the uncoupled oscillators and the noise intensity can be unknown, although the noise intensity could be obtained from the variance of the signal under assumptions of stationarity.

## Results

We first analyze the case with two phase oscillators with time-varying coupling strengths and natural frequencies [48]. This is valid if network interactions are either unknown, or too weak to cause synchronization, and hence assumed to be encompassed in the inherent dynamics of each oscillator, as additive noise and/or non-autonomous (NA) forcing. Then the analysis is extended to homogeneous and delay-imposed networks with bimodal *δ* time delays and log-normally distributed node strengths, as observed in the brain. Analytical findings are numerically validated and are used to explain phase statistics for simulated dynamics over the human connectome.

### Two oscillators

The simplest case of two delay-coupled phase oscillators with constant parameters reads

$$\begin{array}{c} {\dot{\theta }}_{1,2}\left(t\right)=\omega_{1,2}-K\text{sin}\left[\theta_{1,2}\left(t\right)-\theta_{2,1}\left(t-\tau \right)\right]. \end{array}$$ (4)

Steady synchronization occurs when the oscillators start oscillating with a same adjusted frequency Ω, preserving a constant phase shift *ϕ*_1,2_ = *θ*_1_ − *θ*_2_, which becomes

$$\begin{array}{c} \phi_{1,2}=\text{arcsin}\frac{\omega_{1}-\omega_{2}}{2K\;\text{cos}\Omega \tau }\;\in \;\{\begin{array}{cc} (-\frac{\pi }{2},\;\frac{\pi }{2}), & \;\text{if}\;\;K\;\text{cos}\Omega \tau >0,\\ (\frac{\pi }{2},\;\frac{3\pi }{2}), & \;\text{if}\;\;K\;\text{cos}\Omega \tau <0, \end{array} \end{array}$$ (5)

where Ω is described by a transcendental function and the critical coupling reads

$$\begin{array}{c} K_{c}=|\omega_{2}-\omega_{1}|/\left|2\;\text{cos}\Omega \tau \right| \end{array}$$ (6)

Oscillators can be locked in- or anti- phase, depending on the sign of *K* cos Ω*τ*, so that for the phase shift it holds

$$\begin{array}{c} \left\{ \begin{array}{c} \omega_{2}\geq \omega_{1}\;\Rightarrow \;\{\begin{array}{cc} \phi \in [0,\;\frac{\pi }{2})\;\;\; & \;\text{if}\;\;K\;\text{cos}\Omega \tau >0,\;\text{(in-phase),}\;\\ \phi \in (\frac{\pi }{2},\;\pi ]\;\;\; & \;\text{if}\;\;K\;\text{cos}\Omega \tau <0,\;\text{(anti-phase).}\; \end{array}\\ \omega_{2}\leq \omega_{1}\;\Rightarrow \;\{\begin{array}{cc} \phi \in (-\frac{\pi }{2},\;0]\; & \text{if}\;\;K\;\text{cos}\Omega \tau >0,\;\text{(in-phase),}\;\\ \phi \in [\pi ,\;\frac{3\pi }{2})\; & \text{if}\;\;K\;\text{cos}\Omega \tau <0,\;\text{(anti-phase).} \end{array} \end{array}\right. \end{array}$$ (7)

The model is made more realistic by allowing deterministic variability of the frequencies and the coupling, and additive, independent, Gaussian noise

$$\begin{array}{c} {\dot{\theta }}_{1,2}\left(t\right)=\omega_{1,2}+\epsilon_{1,2}\;\text{sin}\;{\hat{\omega }}_{1,2}t-\left(K+\epsilon_{K}\;\text{sin}\;{\hat{\omega }}_{K}t\right)\;\text{sin}\left[\theta_{1,2}\left(t\right)-\theta_{2,1}\left(t-\tau \right)\right]+\eta_{1,2}\left(t\right). \end{array}$$ (8)

**Fig 1:**
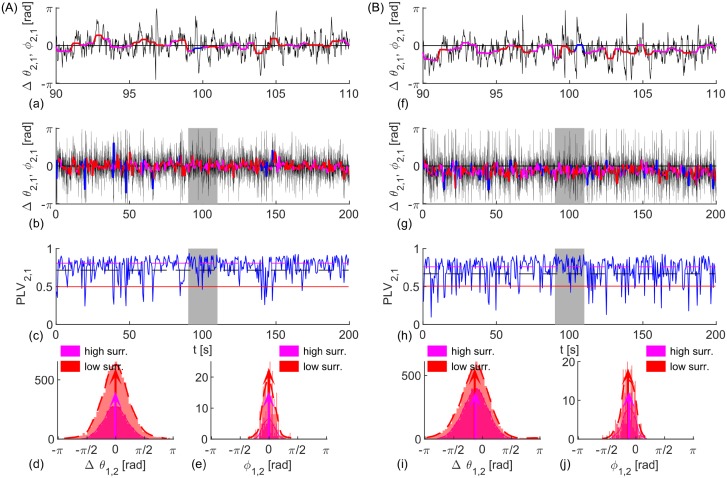
Evolution and statistics of phase metrics for two noisy oscillators with (A) identical and (B) different frequencies. (a, b, f, g) Phase difference, Δ*θ*_1,2_, (black), and angle of the cPLV, *ϕ*_1,2_, (red and magenta for significant PLV and blue otherwise), with the top plots depicting zoomed phases of the shaded area in the lower plots. (c, h) PLV (blue) and its mean value (black), and two levels of significance (magenta and red). (d, e, i, j) PDF (dashed red and full magenta line), histograms of phase differences Δ*θ*_1,2_ and angle *ϕ*_1,2_ during epochs of synchronization, and their circular mean values (red and magenta arrows). Two different surrogates procedures (high surr and low surr) are used for the levels of significance. Parameters: *τ* = 0.01*s*, *D* = 5, *K* = 30 (A) *ω*_1,2_ = 12 ⋅ 2*π* *rad*/*s*; (B) *ω*_1,2_ = 12 ⋅ [0.95, 1.05] ⋅ 2*π* *rad*/*s*.

Here 〈*η*_*i*_(*t*)〉 = 0 and 〈*η*_1_(*t*)*η*_2_(*t*′)〉 = 2*Dδ*(*t* − *t*′)*δ*_1,2_, with 〈 ⋅ 〉 denoting time-average operator, while *ω*_1,2_ and *K* are harmonically modulated. In adiabatic limit without noise [48] effective coupling $K_{eff}\left(t\right)=\left(K+\epsilon_{K}\;\text{sin}\;{\hat{\omega }}_{K}t\right)\text{cos}\Omega \tau$ and frequencies $\omega_{eff1,2}\left(t\right)=\omega_{1,2}+\epsilon_{1,2}\;\text{sin}\;{\hat{\omega }}_{1,2}t$ and Δ*ω*_*eff*1,2_(*t*) = *ω*_*eff*1_(*t*) − *ω*_*eff*2_(*t*), can quantify the synchronization instead of fixed parameters in Eqs (5) and (6), but they give insight into the level of coherence even for stochastic dynamics, and for non-adiabatic response that occurs due to the large inherent time-scale close to incoherence. The stochastic dynamics with constant parameters is shown through the evolution of instantaneous and time-averaged phase lags, and PLVs in Fig 1.

The oscillators in panel (A) are identical and the only variability is due to the noise, which causes time-varying cPLV and phases. Nevertheless, phase lags are close to zero during the periods of significant PLV, as seen by the red and magenta lines for a shorter interval in (a), and for the whole time series in (b). Thus, for a sufficient number of time-points the lags for in-phase synchronization will have a mean at 0, as seen in their histograms and estimated probability density distributions (PDF), (d, e). The mean phase difference in (B) is in the interval (−*π*/2, 0), as predicted by Eq (7) for in-phase locking and different natural frequencies with *ω*_1_ < *ω*_2_. The results also indicate that statistical significance has no influence on the mean of the observed lags, but only impacts their variance. Hence, lower significance levels would improve the statistics of short time-series, by increasing the number of significant data points.

**Fig 2:**
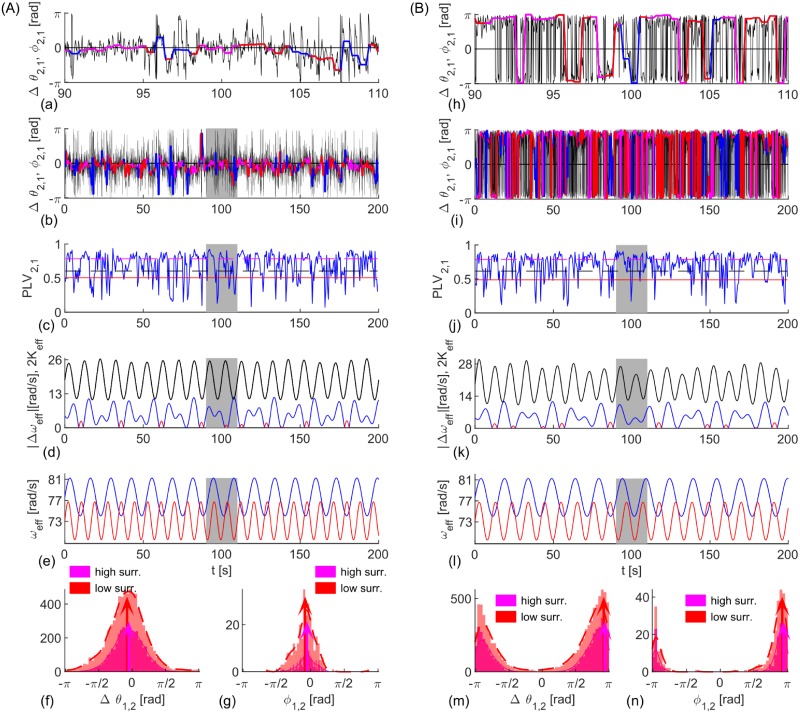
Evolution and statistics of phase metrics for two NA oscillators being (A) in- and (B) anti-phase synchronized. (a, b, h, i) Phase differences Δ*θ*_1,2_(*t*), (black) and *ϕ*_1,2_(*t*), (red and magenta for significant PLV, and blue otherwise) with top plots zooming the shaded interval of the lower plots. (c, j) PLV (blue) and its mean value (black), and levels of significance (magenta and red). (d, k) Effective coupling strength, *K*_*eff*_ (black) compared with the absolute value of the frequency difference, Δ*ω*_*eff*_, (blue if *ω*_1_ < *ω*_2_ and red otherwise), and (e, l) effective natural frequencies (red for *ω*_1_ and blue for *ω*_2_). (f, g, m, n) PDF (dashed red and full magenta line), histograms of phase differences during epochs of synchronization and their means (arrows). Parameters: *ω*_1,2_ = 12 ⋅ [1.03, 0.97] ⋅ 2*π* *rad*/*s*, *ϵ*_1,2_ = [3.6, 3.6], ${\hat{\omega }}_{K}=0.63\;rad/s$, *D* = 5 (A) *τ* = 0.01*s*, ${\hat{\omega }}_{1,2}=\left[0.48,0.74\right]\;rad/s$, *K* = 25, *ϵ*_*K*_ = 5; (B) *τ* = 0.03*s*, ${\hat{\omega }}_{1,2}=\left[0.42,0.60\right]\;rad/s$, *K* = 30, *ϵ*_*K*_ = 6.

Non-autonomicity causes intermittent epochs of in- or anti-phase synchronization, Fig 2. These are still well captured by |2*K*_*eff*_| ≷ |Δ*ω*_*eff*_|, as predicted by Eq (6) for fixed parameters, with periods of insignificant coherence, (c, j), corresponding to |2*K*_*eff*_| ≲ |Δ*ω*_*eff*_|, (d, k). In both examples the coupling is explicitly modulated with ${\hat{\omega }}_{K}$, but also implicitly through the NA frequency of synchronization included in cos Ω(*t*)*τ*, whilst 〈*ω*_*eff*2_〉 is clearly larger than 〈*ω*_*eff*1_〉 for averaging over the times of significant coherence. The latter ensures the phase lags to be in the ranges predicted by Eqs (5) and (7) for *ω*_2_ ≥ *ω*_1_, although they are wider distributed than for fixed parameters, due to the varying frequency mismatch. The distribution of Δ*θ* is additionally broader due to the noise-induced variability, which gets partially averaged out for *ϕ*. As in Fig 1, instantaneous and averaged phases from cPLV have very similar statistics, shown through histograms for significant phase lags in regard to the both levels of significance, calculated at 50 equally spaced bins in the interval [−*π*, *π*], as it is the case in the later figures. This implies that shorter time-windows would affect observed phase shifts only indirectly, through the levels of PLV.

Results for time-varying parameters, Eq (8), in Fig 2 confirm that the theoretical insights, Eqs (5) and (7), can be also used to describe the statistics for noisy and NA parameters, which resemble the intermittent coherence observed in the real data. The distribution of phase lags depends on the time-delay compared with the frequency of synchronization, and the average ratio of the natural frequencies. The former dictates the regime of synchronization, in- or anti-phase, whereas the latter specifies in which quadrant the mean of the phases will be located.

### Networks of oscillators

First we derive analytical results for two spatial configurations of time-delays in all-to-all connected oscillators with heterogeneous natural frequencies and coupling strengths. Then we generalize and numerically validate those results for a more biologically plausible scenario with stochastic inhomogeneities.

The system Eq 2 cannot be solved for general [*K*_*ij*_, *τ*_*ij*_], such as the connectome. Still, based on certain assumptions, we characterize phase relations between different nodes, depending on their location and strength. Firstly we approximate coupling inhomogeneity by the average coupling strength of each oscillator [49], which also allows for sparse networks. The model Eq 2 is henceforth reduced to

$$\begin{array}{c} {\dot{\theta }}_{i}=\omega_{i}+\frac{K_{i}}{N}\sum_{j=1}^{N}\text{sin}\left[\theta_{j}\left(t-\tau_{ij}\right)-\theta_{i}\right],\;\;i=1\ldots N.\;\;\;\; \end{array}$$ (9)

Next, global and local order parameters [13, 36], are defined

$$\begin{array}{ccc} z(t) & \equiv & r\left(t\right){\text{e}}^{i\Phi (t)}=\frac{1}{N}\sum_{j=1}^{N}{\text{e}}^{i\theta_{j}}, \end{array}$$ (10)

$$\begin{array}{ccc} \xi_{i}\left(t\right) & \equiv & R_{i}\left(t\right){\text{e}}^{i\Psi_{i}\left(t\right)}=\frac{1}{N}\sum_{j=1}^{N}{\text{e}}^{i\theta_{j}\left(t-\tau_{ij}\right)}. \end{array}$$ (11)

Here *r* is the global coherence or the strength of the instantaneous mean field, *R*_*i*_ is the local coherence or the mean field strength felt by each oscillator, whilst Φ and Ψ_*i*_ are the phases of the global and the local mean-fields. Introducing these in Eq 9, the mean-field character of the model emerges

$$\begin{array}{c} {\dot{\theta }}_{i}=\omega_{i}+K_{i}\;\text{Im}\left(\xi_{\text{i}}{\text{e}}^{-\text{i}\theta_{\text{i}}}\right) \end{array}$$ (12)

To facilitate the analysis steady partial synchronization [24] is assumed, as opposed to the so-called standing waves [50]. We build on [13] and we derive analytical results for randomly distributed bimodal-*δ* delays and delay-imposed symmetrical biclusters. The PDF of the time delays with equal peaks hence reads

$$\begin{array}{c} h\left(\tau \right)=[\delta \left(\tau -\tau_{1}\right)+\delta \left(\tau -\tau_{2}\right)]/2. \end{array}$$ (13)

**Fig 3:**
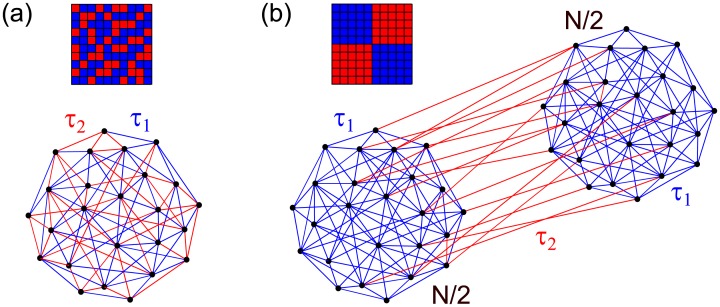
Sketch of spatial distribution of the delays and connectivity matrices. Bimodal *δ* distributed delays with *τ*_1_ (blue) *τ*_2_ (red). (a) Spatially homogeneous (random) delays and (b) heterogeneous, delay-imposed structure.

The delays are either spatially homogeneous with the same independent probability for any link, or they are heterogeneously organized so that two identical subpopulations emerge with same internal and external time-delays, Fig 3. Besides representing distinct phenomenological structures, these topologies are motivated from the connectome. Its simplest decomposition on a left and a right hemispheres identifies the peaks in the delays distribution as intra- and inter- hemispheric links (see Fig 9(d)–9(f)), leading to the clustered organization as a first approximation. However, this division is not strict, and many links are randomly distributed, corresponding to spatial homogeneity.

Due to the spatial homogeneity of the random network and of the internal links of ordered subpopulations, Fig 3, the global order parameter, Eq (10), in both cases is

$$\begin{array}{c} z\left(t\right)=\left(z^{I}+z^{II}\right)/2. \end{array}$$ (14)

For the former *z*^*I*, *II*^ = *z* can represent any proportion of nodes, while for the latter they correspond to the different delay-imposed subpopulations.

#### Bimodal-*δ* spatially homogeneously distributed delays

Because of the spatial homogeneity, steady synchronization for random delays implies

$$\begin{array}{c} r_{i}\left(t\right)=r\left(t\right)=R\left(t\right)=const,\;\;\Phi \left(t\right)=\Omega t, \end{array}$$ (15)

where the initial phase of the mean field is set to 0 without losing generality. This allows relative phases to be introduced, *ϕ*_*i*_ = *θ*_*i*_ − Ω*t*, and considering that contribution of links with different delays can be separated for each oscillator [13], Eq (12) is rewritten as

$$\begin{array}{c} {\dot{\phi }}_{i}=\omega_{i}-\Omega -K_{i}r\;\text{sin}\left(\phi_{i}+\Omega \tilde{\tau }\right)\;\text{cos}\left(-\Omega \Delta \tau \right), \end{array}$$ (16)

where we have introduced $\tilde{\tau }=\frac{\tau_{1}+\tau_{2}}{2},\;\;\Delta \tau =\frac{\tau_{2}-\tau_{1}}{2}>0.$

For steady synchronization fixed point appears in Eq (16), and considering its stability, relative phases read

$$\begin{array}{c} \phi_{i}+\Omega \tilde{\tau }=\text{arcsin}\left(\frac{\omega_{i}-\Omega }{K_{i}r\text{cos}\Omega \Delta \tau }\right)\;\;\in \{\begin{array}{cc} (-\frac{\pi }{2},\;\frac{\pi }{2}), & \;\text{if}\;\;\Omega \Delta \tau \in (-\frac{\pi }{2},\;\frac{\pi }{2}),\\ (\frac{\pi }{2},\;\frac{3\pi }{2}), & \;\text{if}\;\;\Omega \Delta \tau \in (\frac{\pi }{2},\;\frac{3\pi }{2}). \end{array} \end{array}$$ (17)

Thus, ΩΔ*τ* and $\Omega \tilde{\tau }$ are always in the same complex half-plane (left or right), as also shown by the examples in Figs 5 and 6, and the limits of synchronization are defined as

$$\begin{array}{c} K_{i}r\;\text{cos}\;\Omega \Delta \tau >\left|\omega_{i}-\Omega \right| \end{array}$$ (18)

**Fig 4:**
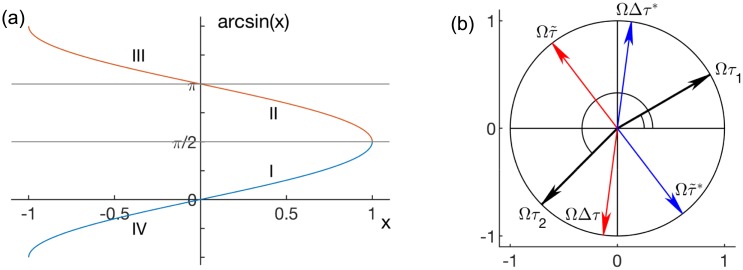
(a) Values of the arcsine function and (b) an example for transformation of ΩΔ*τ* and $\Omega \tilde{\tau }$. (a) Blue for arcsin(*x*) ∈ [−*π*/2, *π*/2] and red for arcsin(*x*) ∈ [*π*/2, 3*π*/2] in the Cartesian plane. (b) Values of Ω*τ*_1,2_ (black) and ΩΔ*τ* and $\Omega \tilde{\tau }$ (red), and ΩΔτ* = ΩΔ*τ* ± *π* and $\Omega {\tilde{\tau }}^{*}=\Omega \tilde{\tau }\pm \pi$ (blue), which appear due to the summation sin(Ω*τ*_1_ + *ϕ*_*i*_) + sin(Ω*τ*_2_ + *ϕ*_*i*_ + 2*π*).

Taking into account the signs of cos ΩΔ*τ* and arcsin(*x*), Fig 4(a), relative phases read

$$\begin{array}{c} \left\{ \begin{array}{cc} \text{if}\;\Omega \Delta \tau \in (\frac{\pi }{2},\;\frac{3\pi }{2}), & \;\phi_{i}+\Omega \tilde{\tau }\in \{\begin{array}{cc} {}[0,\frac{\pi }{2}), & \;\text{for}\;\;\omega_{i}\geq \Omega ,\\ (-\frac{\pi }{2},0], & \;\text{for}\;\;\omega_{i}\leq \Omega . \end{array}\\ \text{if}\;\Omega \Delta \tau \in (-\frac{\pi }{2},\;\frac{\pi }{2}), & \;\phi_{i}+\Omega \tilde{\tau }\in \{\begin{array}{cc} (\frac{\pi }{2},\pi ], & \;\text{for}\;\;\omega_{i}\leq \Omega ,\\ {}[\pi ,\frac{3\pi }{2}), & \;\text{for}\;\;\omega_{i}\geq \Omega . \end{array} \end{array}\right. \end{array}$$ (19)

Moreover, considering the changing of arcsin(*x*) in those ranges, Fig 4(a), the following rules appear for the phases depending on the nodes strengths and frequencies

$$\begin{array}{c} \left\{ \begin{array}{cc} K_{i}↗\;\;\text{and/or}\;\omega_{i}↘\;\;\Leftrightarrow \;\phi_{i}↘ & \;\text{for}\;\;\omega_{i}>\Omega ,\\ K_{i}↗\;\;\text{and/or}\;\omega_{i}↗\;\;\Leftrightarrow \;\phi_{i}↗ & \;\text{for}\;\;\omega_{i}<\Omega , \end{array}\right. \end{array}$$ (20)

where up/down pointing arrows indicate to the increase/decrease of the preceding parameter.

**Fig 5:**
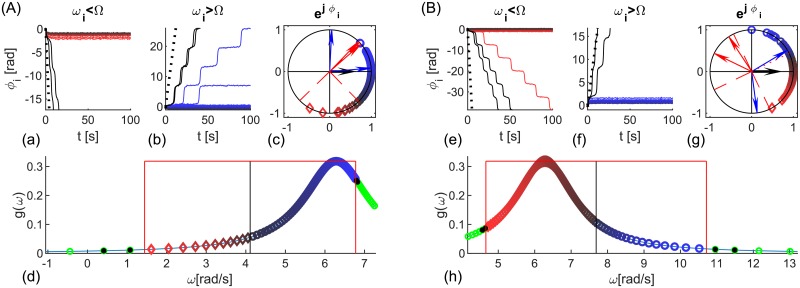
Delay-coupled heterogeneous oscillators with homogeneous bimodal-*δ* delays. Synchronization at frequency (A) Ω < *μ* and (B) Ω > *μ*. (a, b, e, f) Relative phases *ϕ*_*i*_(*t*) of the synchronized and two unsynchronized oscillators (black) closest to the limits Ω ± *Kr* cos ΩΔ*τ*. For comparison ±(Ω − *μ*)*t* are shown with dashed lines. Oscillators with (a, e) *ω*_*i*_ < Ω and (b, f) *ω*_*i*_ > Ω. (c, g) Geometric representation of *ϕ*_*i*_ of the synchronized oscillators (different shades of red diamonds for *ω*_*i*_ < Ω and blue circles for *ω*_*i*_ > Ω) at the end of the simulations. Limits $\pi /2-\Omega \tilde{\tau },\;3\pi /2-\Omega \tilde{\tau }$ and $-\Omega \tilde{\tau }$ are dashed red. The arrows show the complex order parameter (black), angles Ω*τ*_1,2_ (blue), and $\Omega \tilde{\tau }$ and ΩΔ*τ* (red). (d, h) PDF of the natural frequencies, the frequency Ω (black vertical line), and the limits of synchronization Ω ± *rK* cos ΩΔ*τ* (red). Entrained (blue and red) and the first two un-synchronized (black) oscillators are consistent across the plots, and the rest are green. Parameters: Number of oscillators: *N* = 300, Lorentzian natural frequencies with *μ* = 1*Hz* and *γ* = 1 (A) *τ* = [0.02, 0.37]*s*, *K* = 6, (B) *τ* = [0.07, 0.63]*s*, *K* = 8.

**Fig 6:**
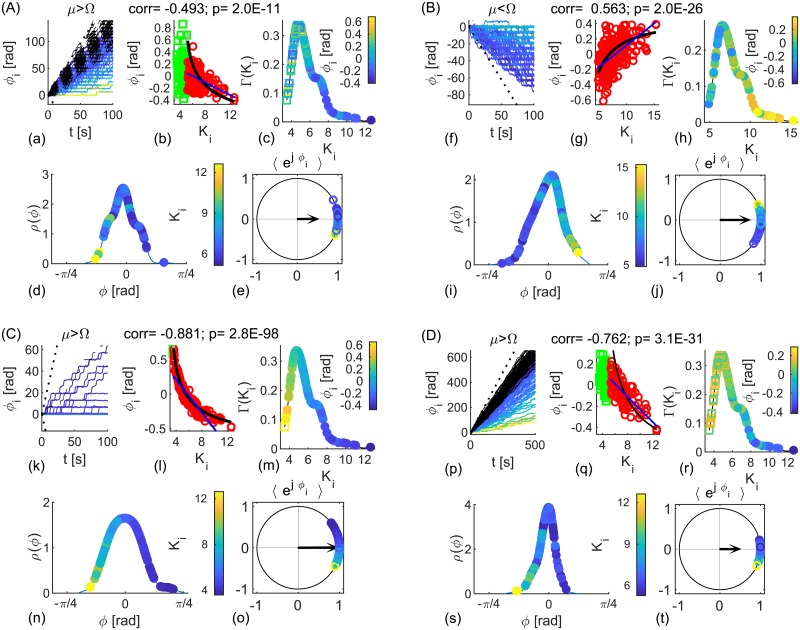
Identical noisy phase oscillators coupled with homogeneous bimodal-*δ* delays and log-normal coupling strengths. Synchronization at frequency (A, C, D) Ω > *μ* and (B) Ω < *μ*. (a, f, k, p) Phases *ϕ*_*i*_(*t*) of the synchronized (colored coded with the in-strength) and unsynchronized (dashed black) oscillators, and the mean phase, ±(Ω − *μ*)*t* (dashed lines). (b, g, l, q) Scatter plot of averaged relative phases and nodes in-strengths, showing synchronized (red) and unsynchronized (green) oscillators. Black line is the theoretical prediction Eq (21) and blue is the linear fit of the correlation. (c, h, m, r) Node strengths color-coded with their phases (filled circles are synchronized, and empty squares are unsynchronized), and their PDF. (d, i, n, s) Phases of synchronized oscillators color-coded with their in-strength, and their PDF, *ρ*(*ϕ*) and (e, j, o t) geometric representation and the complex order parameter (black arrow). Parameters: *N* = 300, simulation time (A-C) *t*_*fin*_ = 200*s* and (D) *t*_*fin*_ = 2000*s*. (A, B, D) *D* = 1, (C) *D* = 0.1. Log-normally distributed in-strengths with *σ*_*K*_ = 2*μ*_*K*_, (A, C, D) *μ*_*K*_ = 6 and (B) *μ*_*K*_ = 8. Delays (A, C, D) *τ* = [0.02, 0.37]*s*, (B) *τ* = [0.07, 0.63]*s*.

Note that for any angles Ω*τ*_1,2_, the values for ΩΔ*τ* and $\Omega \tilde{\tau }$, which appear due to the summation sin(*ϕ*_*i*_ + Ω*τ*_1_) + sin(*ϕ*_*i*_ + Ω*τ*_2_), can be represented on the opposite side of the imaginary axis. This can be shown by adding 2*π* to any of the angles, so that the result of the sum is not changed, while it can be transformed to read

$$\begin{array}{ccc} & & \text{sin}\left(\phi_{i}+\Omega \tau_{1}\right)+\text{sin}\left(\phi_{i}+\Omega \tau_{2}\right)\;=\;\text{sin}\left(\phi_{i}+\Omega \tau_{1}+2\pi \right)+\text{sin}\left(\phi_{i}+\Omega \tau_{2}\right)\;=\\ & & =\;\text{sin}\left(\phi_{i}+\Omega \tau_{1}\right)+\text{sin}\left(\phi_{i}+\Omega \tau_{2}+2\pi \right)\;=\;2\text{sin}\left(\phi_{i}+\Omega \tilde{\tau }+\pi \right)\text{cos}\left(\Omega \Delta \tau +\pi \right). \end{array}$$

Hence, none of the above results will change if both, sin ΩΔ*τ* and cos ΩΔ*τ*, get opposite signs by rotating the angles ΩΔ*τ* and ΩΔ*τ* by *π*, Fig 4(a). In addition, since for stable solutions ΩΔ*τ* and ΩΔ*τ* need to be on the same side of the imaginary axis, only cases when they are in the right half-plane can be considered without any loss of generality.

Numerical results in Fig 5 are for constant couplings, *K*_*i*_ = *K*, and Lorentzian distributed natural frequencies with mean *μ* and scale *γ*

$$\begin{array}{c} g\left(\omega \right)=\gamma /\pi /[{\left(\omega -\mu \right)}^{2}+\gamma^{2}]. \end{array}$$

Regardless of angles $\Omega \tilde{\tau }$ and ΩΔ*τ* being in the right or left complex half-planes, panels (A) and (B), phases are as predicted by Eqs (19) and (20) with slower oscillators being closer to the mean field. Time-evolution of entrained and two closest to them unsynchronized oscillators (a, b, e, f), as well as the position of the phases in the complex plane, (c, g), are shown for the rotating reference frame Ω.

The same populations are simulated in Fig 6, but with equal frequencies and Gaussian noise with a same heterogeneity for the critical coupling for instantaneous interactions [15], i.e. *D* = *γ*. Node’s strengths, *K*_*i*_ = ∑_*j*_ *K*_*ij*_/*N*, are log-normal

$$\begin{array}{c} \Gamma \left(K\right)=\frac{1}{Ks\sqrt{2\pi }}\text{exp}[-\frac{{\left(\text{ln}\;K-m\right)}^{2}}{2s^{2}}],\;\;K>0, \end{array}$$

with mean and variance given as $\mu_{K}=\text{ln}(m/\sqrt{1+\frac{s}{m^{2}}})$, $\sigma_{K}=\sqrt{\text{ln}(1+\frac{s}{m^{2}})}.$ They are assigned to the outgoing links of each node *i*, whereas the symmetric connectivity is enforced by setting *K*_*ij*_ = (*K*_*ij*_ + *K*_*j*,*i*_)/2.

The stochastic frequency heterogeneity averages out over time, hence for the time-averaged phase shifts of the synchronized oscillators we approximately derive

$$\begin{array}{c} \langle \phi_{i}\left(t\right)\rangle +\Omega \tilde{\tau }=\langle \text{arcsin}\left(\frac{\mu -\Omega +\eta_{i}\left(t\right)}{K_{i}r\;\text{cos}\Omega \Delta \tau }\right)\rangle ≊\text{arcsin}\left(\frac{\mu -\Omega }{K_{i}r\;\text{cos}\Omega \Delta \tau }\right). \end{array}$$ (21)

The stability condition is the same as for the deterministic case, while the criterion for synchronization is *K*_*i*_*r* cos ΩΔ*τ* > |*μ* − Ω|. Predictions from Eq (20) still hold, with stronger oscillators lagging for $\Omega \tilde{\tau }$ in the right quadrant, Fig 6(A), and leading otherwise, Fig 6(B). Compared to the Lorentzian frequencies that have an infinite variance, the statistics of noise is homogeneous, but due to the couplings heterogeneity not all oscillators always synchronize, Fig 6(A). Decreasing the noise, while fixing other parameters, Fig 6(c), improves the compliance with the theory, c.f. plots (b) and (l), which is also manifested in the dependency of the phases to the in-strengths, Fig 6(m)–6(o). Similar effect occurs if the simulation time is increased, Fig 6(D), due to the better statistics for longer observations, which averages out the noise.

Time-delays imply multistable solutions for the level and frequency of synchronization, often with no analytical solutions [13, 34, 35]. For all-to-all equally coupled oscillators, low-dimensional evolution of the dynamics [13] reads

$$\begin{array}{ccc} \dot{z} & = & \left(i\mu -\gamma \right)z-K/4[\left(z^{2}\;z_{t-\tau_{1}}^{*}-z_{t-\tau_{1}}\right)+\left(z^{2}z_{t-\tau_{2}}^{*}-z_{t-\tau_{2}}\right)]. \end{array}$$ (22)

From here the frequency of steady synchronization is given as

$$\begin{array}{c} \Omega =\mu -K/2\left(r^{2}+1\right)\text{sin}\left(\Omega \tilde{\tau }\right)\text{cos}\left(-\Omega \Delta \tau \right), \end{array}$$ (23)

Even though this expression is exact only for Lorentzian natural frequencies, it can be used to determine whether the frequency of synchronization is larger or smaller than the natural frequencies, i.e. Ω ≷ *μ*. This inequality depends solely on the signs of $\text{sin}\;\Omega \tilde{\tau }$ and cos ΩΔ*τ*, Eq (23), and governs the general relation of the phase shifts, Eq (21). Hence, taking also into account the stability of *ϕ*_*i*_, Eqs (17) and (21), the behavior of arcsin(*x*), Fig 4(a), and the transformation of sine summation, Fig 4(b), we obtain the directions of change of the relative phases as nodes strength increases, Table 1. All combinations of delays are considered, because phases Ω*τ*_1,2_ might still be longer than a full cycle, despite internal delays being set shorter than the external.

**Table 1:** Change of the relative phases *ϕ*_*i*_ for increasing coupling strengths for spatially random delays.

Ω*τ*_2_	I	II	III	IV
Ω*τ*_1_				
I	↘	unstable ↘	unstable ↗	↗
				↘
II	unstable ↘	unstable	unstable	unstable ↘
III	unstable ↗	unstable	unstable	unstable ↗
IV	↗	unstable ↘	unstable ↗	↗
	↘			

Results are shown for angles Ω*τ*_1,2_ in the different quadrants, during synchronization.

Results from Table 1 can be summarized by the geometrical mean of the time-delays ${\text{e}}^{i\Omega \tilde{\tau }}$, which can be always transformed to be in the right complex half-plane. If ${\text{e}}^{i\Omega \tilde{\tau }}$ or ${\text{e}}^{i(\Omega \tilde{\tau }\pm \pi )}$ is in first quadrant, then Ω < *μ* and stronger nodes phase lag, whilst if it is in the fourth quadrant, then Ω > *μ* and stronger nodes lead. This is confirmed with Fig 6, where the angles are in the same quadrants as for Fig 5.

#### Two clusters with identical bi-modally distributed delays

For fully ordered delay-imposed clusters, the mean field felt by the *i*th oscillator is sum from the delayed mean fields of each population

$$\begin{array}{c} \xi_{i}\left(t\right)=\xi^{I}\left(t\right)+\xi^{II}\left(t\right)=\left[z_{int}\left(t-\tau_{1}\right)+z_{ext}\left(t-\tau_{2}\right)\right]/2, \end{array}$$ (24)

where the subscripts correspond to the mean field from its own (internal), and from the other (external) subpopulation. Assuming steady synchronization with frequency Ω for both clusters, due to the symmetry it follows that

$$\begin{array}{c} R^{I,II}=r^{I,II}=r,\;\Phi^{I,II}=\Omega \left(t-\tau_{1}\right)+\psi_{1,2}, \end{array}$$ (25)

where *ψ*_1,2_ is the phase shift of each cluster and the first is set to zero initial phase without loss of generality, *ψ*_1_ = 0. In [13] it was shown that *ψ*_2_ = 0 ± *π* depending on the sign of cos Ω*τ*_2_, with a stability criterion

$$\begin{array}{c} \psi_{2}=\{\begin{array}{cc} 0, & \;\text{if}\;\;\Omega \tau_{2}\in \left(-\frac{\pi }{2},\;\frac{\pi }{2}\right)+2n\pi ,\\ \pi & \;\text{if}\;\;\Omega \tau_{2}\in \left(\frac{\pi }{2},\;\frac{3\pi }{2}\right)+2n\pi ,\;n=0,1\ldots \end{array} \end{array}$$ (26)

Thus, from Eq (12), synchronized oscillators in the reference frame Ω, Eq (25), follow

$$\begin{array}{c} {\dot{\phi }}_{i}=0=\{\begin{array}{cc} \omega_{i}-\Omega -K_{i}r\;\text{sin}\left(\phi_{i}+\Omega \tilde{\tau }\right)\text{cos}\;\Omega \Delta \tau , & \;\text{if}\;\;\Omega \tau_{2}\in \left(-\frac{\pi }{2},\;\frac{\pi }{2}\right),\\ \omega_{i}-\Omega +K_{i}r\;\text{cos}\left(\phi_{i}+\Omega \tilde{\tau }\right)\text{sin}\;\Omega \Delta \tau , & \;\text{if}\;\;\Omega \tau_{2}\in \left(\frac{\pi }{2},\;\frac{3\pi }{2}\right). \end{array} \end{array}$$ (27)

**Fig 7:**
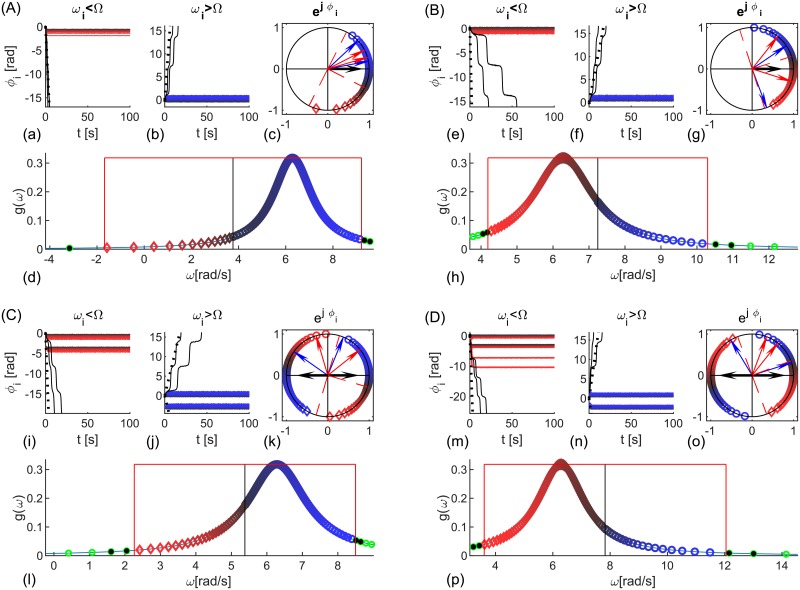
Delay-imposed populations of coupled heterogeneous phase oscillators. (A, B) In- and (C, D) anti- phase synchronized clusters, at frequency (A, C) Ω < *μ* and (B, D) Ω > *μ*. (a, b, e, f, i, j, m, n) Phases *ϕ*_*i*_(*t*) of the synchronized (red and blue) and two unsynchronized oscillators (black) closest to the limits of synchronization, and ±(Ω − *μ*)*t* (dashed). (c, g, k, o) Geometric representation of *ϕ*_*i*_ of the synchronized oscillators (different shades of red diamonds for *ω*_*i*_ < Ω and blue circles for *ω*_*i*_ > Ω). Limits $\pi /2-\Omega \tilde{\tau },\;3\pi /2-\Omega \tilde{\tau }$ and $-\Omega \tilde{\tau }$ are dashed red, the arrows are for the complex order parameter (black) of each subpopulation (they overlap for in-phase), angles Ω*τ*_1,2_ (blue), and $\Omega \tilde{\tau }$ and ΩΔ*τ* (red). (d, h, l, p) PDF of the natural frequencies, frequency of synchronization Ω (black vertical line), and limits of synchronization (red). Entrained oscillators are blue and red, the first two un-synchronized on both sides are black, and the rest are green. The colors of each oscillator are consistent across the plots. Parameters: *N* = 300, *K* = 7, Lorentzian natural frequencies with *μ* = 1*Hz* and *γ* = 1. (A) *τ* = [0.05, 0.2]*s*, (B) *τ* = [0.7, 0.95]*s*, (C) *τ* = [0.22, 0.47]*s*, and (D) *τ* = [0.04, 0.27]*s*.

**In phase synchronization**. Stable in-phase solutions of Eq (27) are identical to the case of spatially homogeneous delays, Eq (17), with the same condition for synchronization, Eq (18). Since these are stable only for ${\text{e}}^{i\Omega \tilde{\tau }}$ and *e*^*i*ΩΔ*τ*^ on the same side of the imaginary axis, the transformation of the sine sum and the properties of arcsin(*x*), lead to

$$\begin{array}{c} \left\{ \begin{array}{cc} \omega_{i}>\Omega \;\Leftrightarrow \phi_{i}+\Omega \tilde{\tau }\in \left(0,\frac{\pi }{2}\right) & \;\;\Rightarrow \;K_{i}↗\;\;\text{and/or}\;\omega_{i}↘\;\;\Leftrightarrow \;\phi_{i}↘\\ \omega_{i}<\Omega \;\Leftrightarrow \phi_{i}+\Omega \tilde{\tau }\in \left(-\frac{\pi }{2},0\right), & \;\;\Rightarrow \;K_{i}↗\;\;\text{and/or}\;\omega_{i}↗\;\;\Leftrightarrow \;\phi_{i}↗, \end{array}\right. \end{array}$$ (28)

which is the same relation as for spatially random delays, Table 1. These results are numerically confirmed in Fig 7(A) and 7(B), where for identical coupling strengths, faster oscillators phase lead the slower ones. The limits of synchronization are also confirmed, as well as the behavior of the phases for *ω*_*i*_ ≷ Ω as predicted by Eq (28).

**Anti phase synchronization**. For Ω*τ*_2_ in the left complex half-plane, clusters are anti-phase locked with mean fields at distance *π* and same frequency and level of synchronization. Relative phases are the stable solutions of Eq (27), which read

$$\begin{array}{c} \phi_{i}+\Omega \tilde{\tau }=\text{arccos}\left(-\frac{\omega_{i}-\Omega }{K_{i}r\;\text{sin}\Omega \Delta \tau }\right)\;\;\in \{\begin{array}{cc} (0,\;\pi ), & \;\text{if}\;\;\Omega \Delta \tau \in (-\frac{\pi }{2},\;\frac{\pi }{2}),\\ (\pi ,\;2\pi ), & \;\text{if}\;\;\Omega \Delta \tau \in (\frac{\pi }{2},\;\frac{3\pi }{2}), \end{array} \end{array}$$ (29)

whilst the criterion for entrainment of single oscillators is *K*_*i*_*r*|sin ΩΔ*τ*| > |*μ* − Ω|. As before, transforming ${\text{e}}^{i\Omega \tilde{\tau }}$ and *e*^*i*ΩΔ*τ*^ to be in the right half-plane, without any loss of generality we focus only on the interval (0, *π*), yielding

$$\begin{array}{c} \left\{ \begin{array}{cc} \omega_{i}>\Omega \;\Leftrightarrow \;\phi_{i}+\Omega \tilde{\tau }\in \left(\frac{\pi }{2},\pi \right), & \;\;\Rightarrow \;K_{i}↗\;\;\text{and/or}\;\omega_{i}↘\;\;\Leftrightarrow \;\phi_{i}↘,\\ \omega_{i}<\Omega \;\Leftrightarrow \;\phi_{i}+\Omega \tilde{\tau }\in \left(0,\frac{\pi }{2}\right), & \;\;\Rightarrow \;K_{i}↗\;\;\text{and/or}\;\omega_{i}↗\;\;\Leftrightarrow \;\phi_{i}↗. \end{array}\right. \end{array}$$ (30)

**Fig 8:**
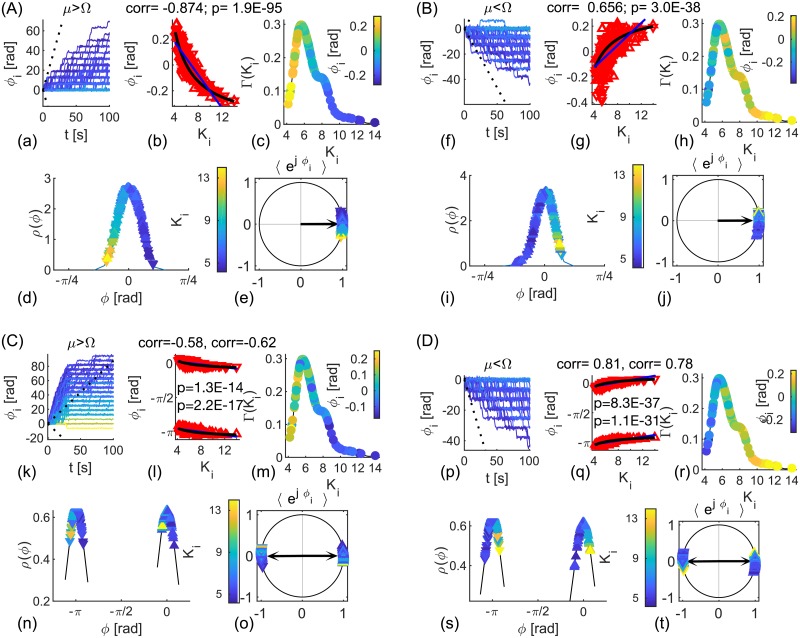
Delay-imposed clusters of identical noisy phase oscillators. (A, B) In- and (C, D) anti- phase synchronization, with log-normally distributed coupling strengths *K*_*ij*_. (a, f, k, p) Phases *ϕ*_*i*_(*t*) of synchronized oscillators (color-coded with their in-strength) and the mean phase, ±(Ω − *μ*)*t* (dashed). (b, g, l, k) Scatter plot of averaged relative phases and nodes strength. Oscillators of the different populations are with opposite pointing triangles. Black line is the theoretical prediction, Eq (33), blue is the linear fit for each population. (c, h, m, r) Node strengths color-coded with their relative phases and their PDF. (d, i, n, s) Phases of the synchronized oscillators (color-coded with their in-strength) and their PDF, and (e, j, o, t) their geometric representation and complex order parameter (black arrow). Parameters: *N* = 300, *K* = 7, *D* = 1. (A) *τ* = [0.05, 0.2]*s*, (B) *τ* = [0.7, 0.95]*s*, (C) *τ* = [0.22, 0.47]*s*, and (D) *τ* = [0.04, 0.27]*s*.

Hence, the phase shifts within the same cluster have the same dependence from the sign of *ω*_*i*_ − Ω, as for in-phase synchronization, Figs 7 and 8.

Next we analyze the sign of *μ* − Ω, which for deterministic bell-shaped frequency distribution or for stochastic frequencies implies the sign of *ω*_*i*_ − Ω for a large majority of oscillators. For all-to-all equal couplings and Lorentzian frequencies [13], the low-dimensional dynamics reads

$$\begin{array}{c} {\dot{z}}^{I,II}=\left(i\mu -\gamma \right)z^{I,II}-K/4\left[\left(z^{I,II\;2}\;z_{t-\tau_{1}}^{I,II\;*}-z_{t-\tau_{1}}^{I,II}\right)+\left(z^{I,II\;2}z_{t-\tau_{2}}^{II,I\;*}-z_{t-\tau_{2}}^{II,I}\right)\right]. \end{array}$$ (31)

Taking also into account Eq (25), the frequency of steady synchronization becomes

$$\begin{array}{c} \Omega =\{\begin{array}{cc} \mu -\frac{K}{2}\left(r^{2}+1\right)\;\text{sin}\Omega \tilde{\tau }\;\text{cos}\Omega \Delta \tau , & \;\text{if}\;\;\Omega \tau_{2}\in \left(-\frac{\pi }{2},\;\frac{\pi }{2}\right),\\ \mu +\frac{K}{2}\left(r^{2}+1\right)\;\text{cos}\Omega \tilde{\tau }\;\text{sin}\Omega \Delta \tau , & \;\text{if}\;\;\Omega \tau_{2}\in \left(\frac{\pi }{2},\;\frac{3\pi }{2}\right). \end{array} \end{array}$$ (32)

This allows evaluating increase/decrease of phases *ϕ*_*i*_ as function of the nodes strength *K*_*i*_ in Eq (27). Results for in- and anti-phase synchronization, Eqs (28) and (30), are summarized in Table 2. Note again that node-strength dependent change of the phases is identical for both models, Tables 1 and 2, when Ω*τ*_2_ is in the right complex half-plane.

**Table 2:** Change of the relative phases *ϕ*_*i*_ for increasing coupling strengths for delay-imposed structure.

Ω*τ*_2_	I	II	III	IV
Ω*τ*_1_				
I	↘	↗	↘	↗
		↘		↘
II	unstable ↘	unstable ↘	unstable ↘	unstable ↘
III	unstable ↗	unstable ↗	unstable ↘	unstable ↗
IV	↗	↗	↗	↗
	↘		↘	

Results are shown for angles Ω*τ*_1,2_ in the different quadrants, during in and anti-phase synchronization.

To compare nodes from different hemispheres during anti-phase regime, the *π* shift between the mean phases need to be considered in relations in Eqs (27) and (30). Henceforth, due to the periodicity of phases, the nodes whose phases lag the most in one cluster, will be closest to the leading nodes in the other. Similarly, for *ω*_*i*_ > Ω stronger and slower nodes will be further away from the mean of the opposite cluster, although they will be closer to their own mean phase. This is illustrated in Fig 7(C) and 7(D) with nodes of the opposite ends in the frequency spectrum for each population being closest to each other—light red and light blue nodes are furthest apart within, but closest to each other between clusters.

Phases for distributed coupling strengths and stochastic inhomogeneities read

$$\begin{array}{c} \langle \phi_{i}+\Omega \tilde{\tau }\rangle =\{\begin{array}{cc} \langle \text{arcsin}\left(-\frac{\mu -\Omega +\xi_{i}\left(t\right)}{K_{i}r\;\text{cos}\Omega \Delta \tau }\right)\rangle ≊\text{arcsin}\left(-\frac{\mu -\Omega }{K_{i}r\;\text{cos}\Omega \Delta \tau }\right), & \;\text{if}\;\Omega \tau_{2}\in \left(-\frac{\pi }{2},\;\frac{\pi }{2}\right),\\ \langle \text{arccos}\left(\frac{\mu -\Omega +\xi_{i}\left(t\right)}{K_{i}r\;\text{sin}\Omega \Delta \tau }\right)\rangle ≊\text{arccos}\left(\frac{\mu -\Omega }{K_{i}r\;\text{sin}\Omega \Delta \tau }\right), & \;\text{if}\;\Omega \tau_{2}\in \left(\frac{\pi }{2},\;\frac{3\pi }{2}\right), \end{array} \end{array}$$ (33)

with a same stability criterion as for deterministic case, while synchronization limits accordingly become *K*_*i*_*r*|cos ΩΔ*τ*| > |*μ* − Ω| and *K*_*i*_*r*|sin ΩΔ*τ*| > |*μ* − Ω|. Plots in Fig 8, show the same examples as in Fig 7, but with stochastic frequency inhomogeneity and log-normally distributed coupling strengths that dictate phase offsets. As predicted by Eqs (28) and (30), within the same population stronger nodes phase lag the weaker for *μ* > Ω, and otherwise. For the nodes belonging to different clusters during anti-phase regime, periodicity causes weaker nodes of one cluster to be closer to stronger nodes of the other, Fig 8(n), 8(o), 8(s) and 8(t).

Results for both types of networks, Figs 6 and 8, indicate that only if both delays are in the first quadrant, stronger brain regions will always phase-lag behind the weaker ones, in agreement with [22]. For this also the natural frequencies of each region are supposed to be equal on average, so that they will be all locked at a lower frequency than their natural. Hence, assuming realistic range of conduction velocities, this would hold only for lower bands of the EEG frequencies.

The above analysis and the results for bimodal *δ* time-delays, homogeneous or clustered, can be generalized for any multimodal *δ* distributed delays, or for combination of random and clustered delays. However these are not supposed to bring qualitatively new types of steady synchronization, while making the analysis more cumbersome.

### Connectome based modeling (numerical results)

General analysis for networks with time-delays is practically impossible to this date. Analytic approaches exist only for certain types of complex networks [16] combined with special delay heterogeneities [13, 36], but they are still limited to the thermodynamic limit or require averaging. Besides, the connectome typically consists of less than 100 nodes, rarely going above several hundreds, and the state of art large-scale brain-modeling considers personalized connectomes [51]. Therefore, numerical simulations scrutinized by the analytical insights for simpler network topologies are a reasonable direction to proceed with the analysis of the brain networks dynamics.

**Fig 9:**
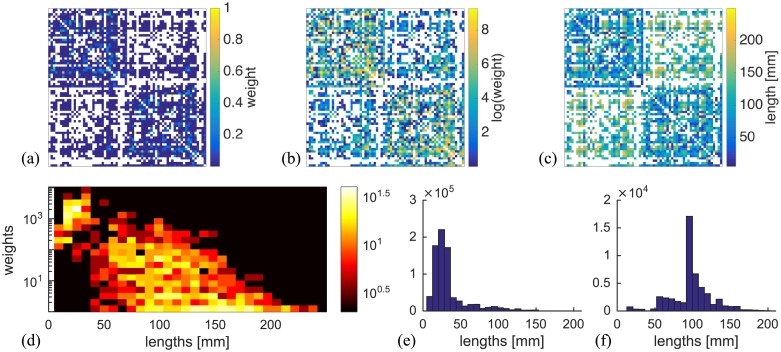
Connectome of a healthy subject. (a) Normalized weights, (b) logarithmic weights and (c) lengths of the tracks. (d) Joint distribution of weights and lengths, and histograms of weighted lengths for (e) intra- and (f) inter-hemisphere links.

In-silico oscillatory neural activity is explored over connectome based architecture to better understand the phase relation between signals from distant brain areas. A human connectome, Fig 9, is randomly chosen from a list of 1200 publicly available healthy subjects part of the Human Connectome project [52]. The subject was scanned on a customized 3 *T* scanner at Washington University and the structural connectivity was constructed using a publicly available pipeline [53] that applies spherical deconvolution method to a probabilistic streamlines tracking algorithm [54]. The obtained connectome consists of few million tracts spatially averaged to connect 68 cortical regions defined according to Desikan-Kiliany atlas [55]. Note however, that different parcellations are also possible, for example by subdividing each of the cortical regions [56], and these can consist of several thousand nodes [57], but are not commonly used in simulations because of the computational cost.

For each link, weights are numbers of individual tracts, Fig 9(a), and lengths are their averages, Fig 9(b). Spatial distribution of the track lengths show that they are bimodaly distributed, Fig 9(d), with the modes being spatially heterogeneous, and as a first approximation corresponding to the intra- and inter-hemispheric links, Fig 9(e) and 9(f). This insight suggests that some of the aspects of the large-scale brain dynamics are expected to be explained by the results for fully ordered delays.

The propagation velocity is fixed within the realistic range [2, 58] at 5*m*/*s*, and dynamics are analyzed at different frequencies and coherence levels. The latter are additionally constrained by the noise and the global coupling strength that multiplies the normalized weights of the connectome. Since the distribution of natural frequencies across brain regions is generally unknown, equal values with stochastic inhomogeneities are assumed at each node. Moreover, even band-pass filtered recordings of neural activity in most of the cases consist of several overlapping rhythms, which are time-varying and activity-dependent, henceforth equal on average for long recordings.

Time delays cause coexistence of multiple stable frequencies of synchronization, larger or smaller than the natural, and can lead to amplitude and oscillation death in more complex systems [59]. However, we observe that unlike for the networks with bi-modally distributed delays, all numerical simulations on the connectome evolve towards a state with lower frequency than the natural, as often reported for different configurations of delay-coupled phase oscillators [60–63].

**Fig 10:**
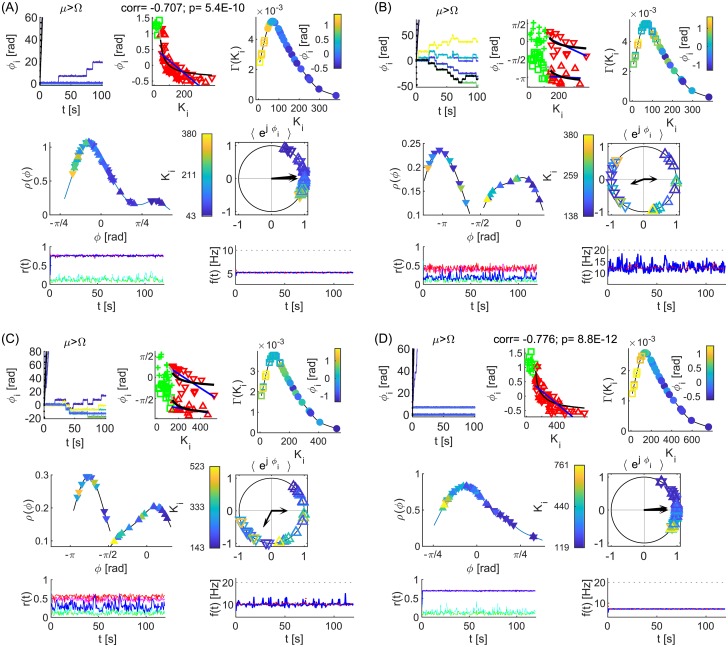
Simulated dynamics over a healthy human connectome. In-phase (A, D) anti-phase (B) and intermittent synchronization (C). Top left plot of each panel are relative phases for the synchronized and two unsynchronized oscillators (black) closest to the limits, and ±(Ω − *μ*)*t* (dashed). Top middle are scatter plots of nodes averaged phases versus their in-strengths. Nodes of left/right hemisphere are up/down pointing triangles, black line is a theoretical prediction, blue is the linear fit. Top right are the PDF of in-strengths color-coded with nodes’s phases. Middle left are phases of the synchronized oscillators (color-coded with in-strength) and their PDF; and middle right is their geometric representation and complex order parameters (black arrows). Bottom left and right are evolutions of order parameter and mean field frequencies, for whole brain (blue) and for each hemisphere (red and magenta). Order parameters for uncoupled case are green for whole brain and cyan for one hemisphere. Parameters: *N* = 68 oscillators, noise intensity *D* = 2. Coupling strengths (A, B) *K* = 0.8, (C) *K* = 1.1, (D) *K* = 1.6, natural frequency (A) *f* = 10*Hz* and (B–D) *f* = 20*Hz*.

**Pair-wise phase lags**. Even though the spatio-temporal structure of the connectome is far more complex than networks with bi-modal *δ* time-delays, results in Fig 10 still show in- (A, D) and anti-phase (B) clusterings between the brain hemispheres for realistic frequencies and different levels of synchronization. An intermittent state of in and anti-phase epochs is also often observed, panel (C), as seen by the mean-field parameters shown on the bottom. If the frequency is such that *μ*〈*τ*_*ext*_〉 is in the right hemisphere, then the latter regimes occurs for most of the cases with low coherence. High coherence almost exclusively leads to slowing down that pushes Ω〈*τ*_*ext*_〉 in the first quadrant and therefore in-phase synchronization. Nevertheless such levels of coherence are not expected to occur in a healthy brain, and these regimes are biologically implausible, Fig 10(A) and 10(D).

The good match of the simulated brain dynamics with the theoretical predictions for networks with much simpler structure, is not only limited to the regimes of synchronization. As predicted, in all examples in Fig 10 stronger nodes in each hemisphere generally lag in phase, since Ω < *μ*. This occurs for in- and anti-phase synchronization, but also during the intermittent regime. The division between the latter two is often fuzzy, since the intervals of anti-phase synchronization rarely last longer than several seconds, before being interrupted with in-phase epochs.

**Fig 11:**
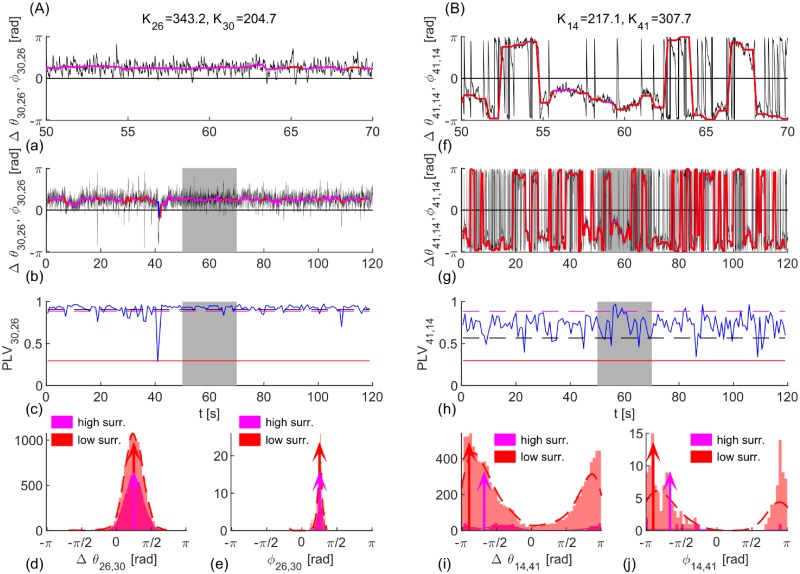
Evolution and statistics of PLV and phase lags for one (A) intra and (B) inter-hemispheric link. (a, b, f, g) Phase lag, Δ*θ*, (black), and angle of the cPLV, *ϕ*, (red and magenta for significant PLV, blue otherwise). (c, h) PLV (blue) and its mean value (black), and two levels of significance (magenta and red). (d, e, i, j) Estimated PDF (magenta line and dashed red) and histograms of phase lags, and their means (red and magenta arrows). Parameters: *f* = 20*Hz*, *D* = 2, (A) *K* = 1.1, (B) *K* = 1.16.

Anti-phase regime is assumed when the hemispheric complex order parameters are at a distance larger than *π*/2, and in-phase otherwise, allowing comparison with the analytical results. They capture the dynamics fairly well, even for a distance not much larger than *π*/2 as shown in Fig 10(C). A better approach would be intermittent intervals to be analyzed separately, since the frequency of synchronization might differ during each interval, and averaging it can lead to wrong values for the phases. Moreover, even if varying frequencies of synchronization are properly detected, averaging of the relative phases over different regimes, Fig 10(B) and 10(C), makes them to be distributed at distances smaller than *π*, which might be mistaken for an actual stationary clustering, rather than a mix of 0 and *π* clusterings. This is shown in Fig 11, where PLV and the instantaneous and time-averaged phase lags are shown for an intra and an inter hemispheric links.

The left panel of Fig 11 shows an intrahemispheric link between in-phase brain regions, and the right depicts an interhemispheric link with epochs of in- and anti-phase locking. Since *K*_26_ > *K*_30_ the phase difference Δ*ϕ*_30,26_ ∈ [0, *π*/2) and results are very similar for the both significant levels, despite their large difference. The region 41 is stronger connected than the 14, so it is expected that during in-phase intervals Δ*ϕ*_14,41_ ∈ (−*π*/2, 0], and Δ*θ*_14,41_ ∈ (*π*/2, *π*] for anti-phase, c.f. with Fig 8(n) and 8(o). Consequently, distributed peaks appear for the histograms of phase lags in the bottom plots of Fig 11(B), but their mean is in (−*π*/2, −*π*] leading to possible wrong conclusion about the synchronization of these nodes.

**Fig 12:**
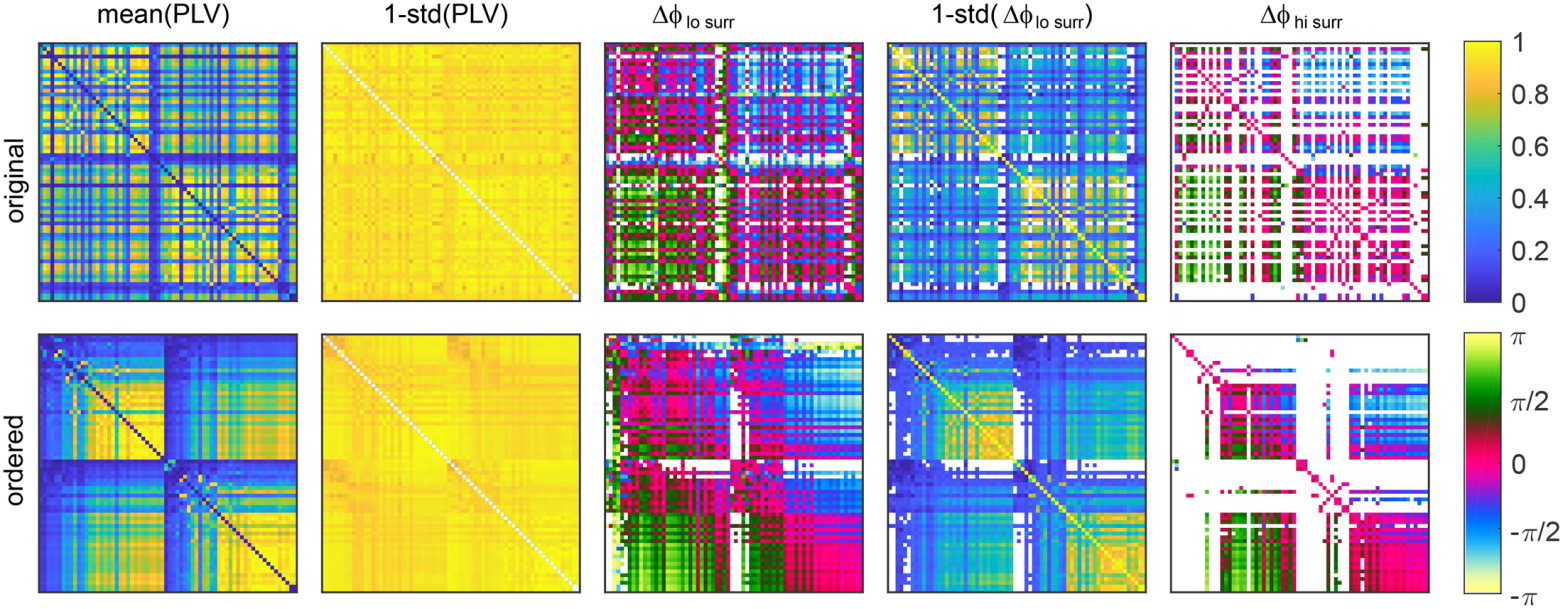
Statistics of PLV and phases for 68 brain regions. Nodes are as given by Desikan Kiliany parcelation (top) and ordered within hemispheres by the in-strength (bottom). For each link the mean and 1–standard deviation are shown, while white are links with no periods of significant coherence. Parameters: *K* = 1.18, *f* = 20*Hz*, *D* = 2.

**Whole brain phase lags statistics**. Whole brain phase statistics are characterized by the mean and the standard deviation of the PLVs, and the correspondent phase-lags for each pair of brain regions. These are shown in Fig 12, where 1–standard deviation is plotted to keep the colors/coherence consistency across the images. In the upper row, the regions are arranged according to Desikan-Kiliany atlas [55], with the left hemisphere first, while in the lower row, nodes of each hemisphere are ordered increasingly according to their strength.

Strengths of the tracts are reflected in PLV (first column in Fig 12), where the links with stronger direct connection show higher functional connectivity. The negative bias of the tracking techniques towards interhemispheric connections is also manifested. Consequently fewer external links have significant coherence, especially for the higher surrogates criterion, when only the strongest links survive. Phase lags within hemispheres, especially for stronger links, are around 0, revealing the in-phase synchronization. Between hemispheres, the phase lags are less informative, due to the intermittent in- and anti-phase synchronization, also seen in Fig 11(B) for the same regime. Still, many inter-hemispheric links have phases around ±*π* or closer to ±*π*/2. The latter is often hallmark of intermittent in and anti-phase regimes, as discussed for the results of Fig 11(B). The intermittency is also manifested by increased variance of the phases, visible for the inter-hemispheric links. This is much less manifested in the coherence, which stays stable during different regimes, as was also indicated by the hemispheric order parameters in Fig 10. The large variation of the overall order parameter observed there, is only due to the bursts of anti-phase ordering when it gets close to 0, whereas the coherence within each hemisphere is quite stable.

**Fig 13:**
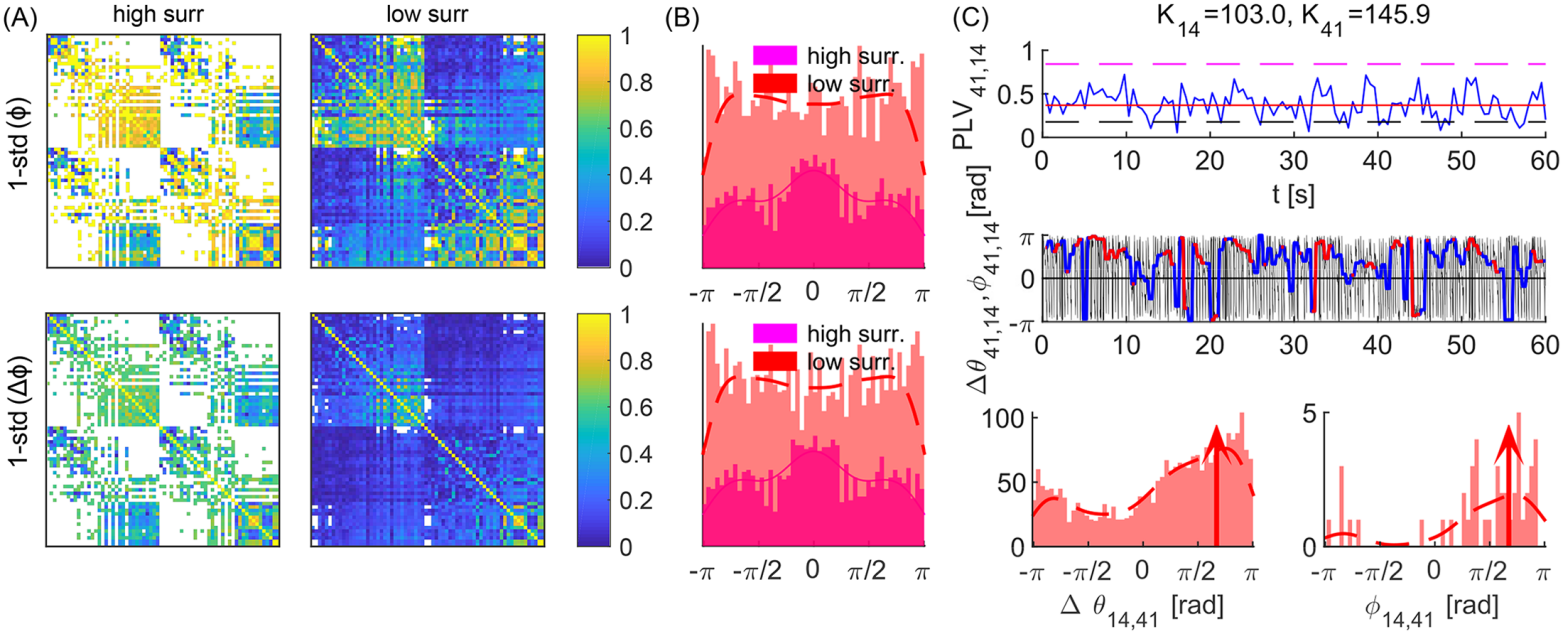
Statistics of PLV metrics for two significance levels. (A) Standard deviation of phase lags. (B) Histogram of mean phase lags for all the links, calculated over periods of significance. (C) Evolutions of PLV (top) and phase lags (middle), and their histograms (bottom) for both levels of significance (magenta and red in all plots) for one inter-hemispheric link. Parameters: *K* = 0.55, *f* = 20*Hz*, *D* = 4.

The impact of the chosen significant coherence, and the difference between instantaneous and averaged phases for the phase statistics of each link is illustrated in Fig 13 for anti-phase regime. Higher significance level causes only slightly larger variance for the phase lags, panel (A), but as seen in Fig 11, it can substantially reduce the number of accounted links, especially between hemispheres, illustrated through one such a link in panel (C). It is due to the latter mechanism that the overall distribution of the mean lags is less uniform for higher surrogates, panel (B). On contrary, time-averaging stronger decreases the variance for the links, because it diminishes the network and stochastic heterogeneities, but it does not affect the means of the phase lags for particular links, as can be also seen for the link in Fig 13(C) or in Fig 11(B).

**Fig 14:**
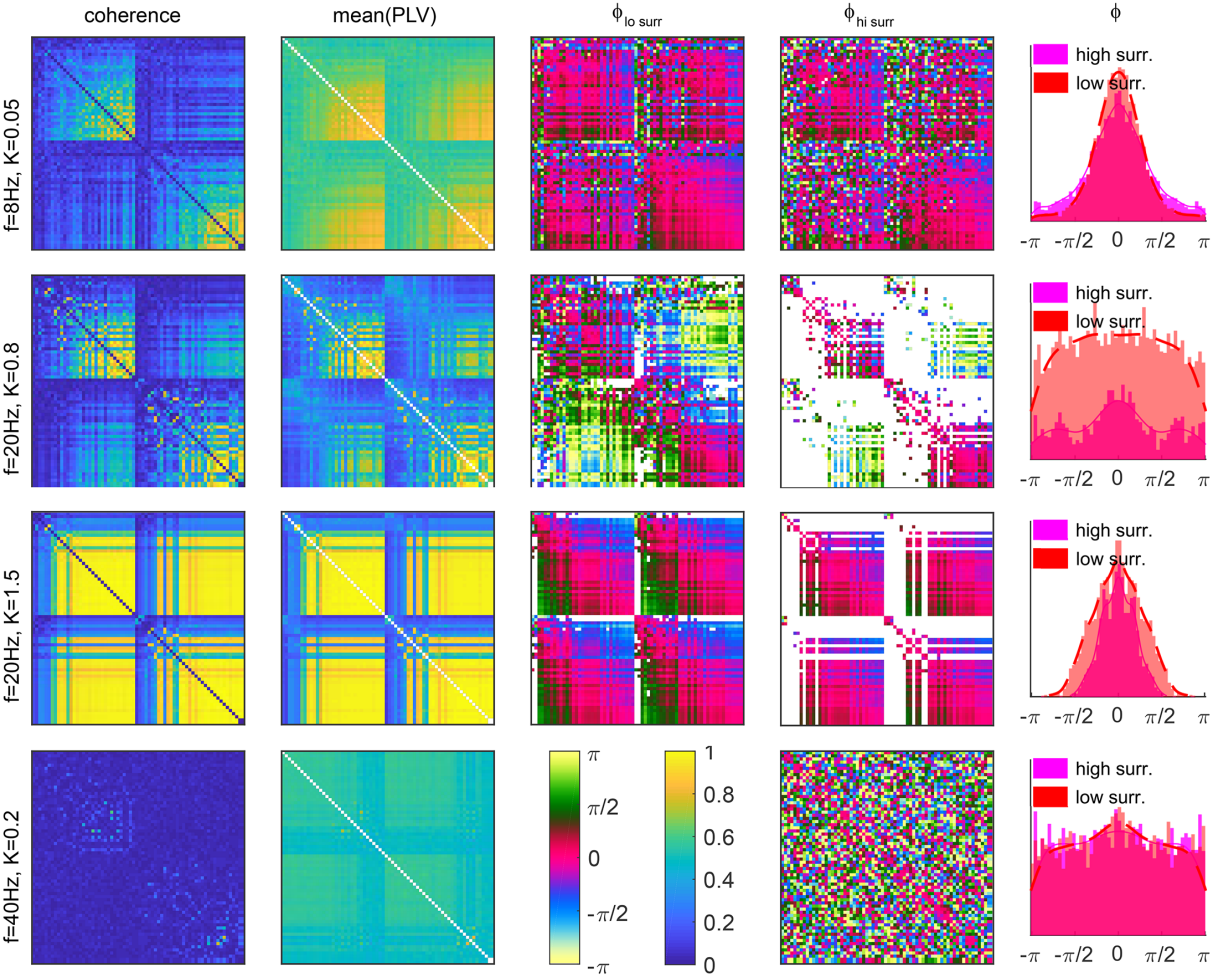
PLV and phase lags during different regimes of brain dynamics. (left to right) Coherence over the whole time-series, mean PLV, mean phase lags *ϕ* for low and high coherence (they are identical for the bottom row), and histograms and PDF of the mean phase lags of coherent links. White are the links with no significant PLV. Nodes in all matrices are sorted by their in-strength. The level of the noise is *D* = 0.2*f*.

The overall statistics for the distinctive phase regimes in the brain are illustrated in Fig 14. Phase lags and PLVs are depicted for a same subject, for various frequencies and coupling strengths, with noise proportional to the frequency to account for the frequency dependent decrease of the coherence [47]. Time-averaged coherence is shown in the first column, in addition to the mean of the PLVs of each time-window. The former speaks about the overall regime of synchronization, whilst the latter depends on the length of windows compared to the frequency and is more affected by the noise. The mean PLV alone is hence not informative, but needs to be compared with a significance level. Mean phase lags for times of significant coherence are shown in the third and fourth column for two different surrogate procedures. They produce largest difference for anti-phase regime (second row), which requires low coherence that is even smaller between hemispheres due to fewer tracts. For very low synchronization, as shown on the bottom, they are identical and therefore only one is shown, while for high overall coherence (second and third row), higher significance level discards the tails in phase lags’ distribution (last column), which mainly represent links between weaker nodes, thus making the distribution sharper.

Possible paths for transition between different regimes of synchronization are also shown in Fig 14. For low frequencies, in-phase synchronization occurs (first row), which becomes intermittent/anti-phase for increased frequency (second row), at similar level of overall coherence. By increasing the coupling and henceforth the level of coherence, the brain switches to in-phase regime (third row) that can again switch to anti-phase by further increasing the frequency, but only at very low overall coherence (bottom row). Also note that the overall low coherence at the bottom row leads to spatially homogeneous values for the mean PLVs, as compared to the cases with much higher coupling and global partial coherence shown in the second and third row. Low coupling renders all links to be around a same level of coherence, without strongly coherent and incoherent links like in the middle rows, and as a result, for every pair of regions exists at least one time-window with statistically significant PLV. Henceforth the absence of links with no significant PLV.

Spatial distribution of phase-lags in Fig 14 is in agreement with the theoretical predictions. Besides being 0 centered for strong nodes within the same hemisphere regardless of the synchronization regime, lags around 0 and ±*π* appear between the hemispheres, resembling in- and anti-phase regimes. In addition, weak regions lead the stronger, and for anti-phase hemispheres *π* is added. Hence, the inverted distribution of green and blue shades for the intra and inter-hemispheric links in the phase lags matrices, with darker shades corresponding to ±*π*/4 for internal links, and lighter for external with the values around *π* ± *π*/4.

**Fig 15:**
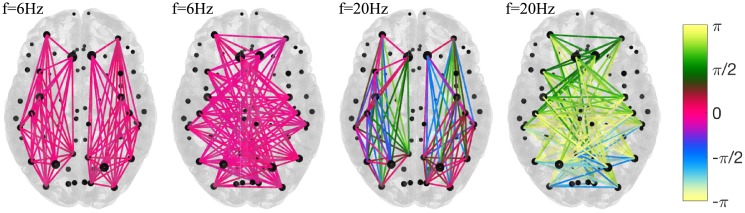
Intra- and inter- hemispheric subnetworks of the 10 strongest nodes. In- and anti-phase synchronization. Parameters: *K* = 0.5, *D* = 0.5.

Frequency dependent spatial distribution of phase lags is illustrated in Fig 15 for intra and inter-hemispheric brain subnetworks, for two frequencies and a same global coupling. The subnetworks consist of the 10 strongest brain regions in each hemisphere based on the sum of their outgoing links. Strength of the nodes is reflected in their size, whilst links are color-coded with their phase lags taken from the upper triangle of the matrices. As predicted, strong coherence is observed during in-phase synchronization at *f* = 6*Hz*, which together with similar strengths of the nodes, renders almost zero phase lags for all the links, internal and external. During anti-phase regime observed at 20*Hz*, the links within the hemispheres have lags distributed around 0, but much wider than before, whilst those between them are distributed around ±*π*. The coherence decreases for increasing frequencies, and together with the earlier discussed non-stationarity, these cause far higher variability of phases, than during in-phase synchronization. Hence the appearance of dark shades of green and blue for in-phase, and light for anti-phase synchronization.

## Discussion

In this study we analyze the mechanisms by which the spatio-temporal features of the connectome impose the architecture of phase lags between distant brain regions. A general relationship is provided for how organization of time-delays drives the hemispheres to in or anti phase coherence, whereas the topology dictates the sign of the phase lags. Both aspects of connectome also determine the overall coherence, which restricts the regimes of phase organization that can be observed. The presented qualitative findings are relevant for phases in frequency decomposed neural activity.

### Model set-up

Phase lags are analyzed when only pair-wise interactions are explicitly considered, and for network connectivity. The former approach is justified if interactions are too weak, and can be represented on average as a stochastic influence to the inherent dynamics at each region. This leads to mean value of the phase differences at 0 or ±*π*, depending on whether the delay is long enough to change the sign of the interaction.

Despite its simplicity that allows analytical tractability, the phenomenological oscillatory model resembles the non-stationary oscillations of the neural activity, which is characterized by transient synchronization. To better understand the underlying organization that regulates the large-scale brain dynamics, and henceforth the phase relationships between network nodes, we analyze synchronization for networks with bimodal delays as a first approximation of the connectome. Theoretical insights are validated numerically for more realistic frequency and couplings heterogeneities, and compared with in-silico brain dynamics, while examining the methodological limitations.

Phase lags during these epochs of coherence depend on the delays, which are constant, and on coupling strength and frequency mismatch. The latter can be different across the time-series, but the statistics of the narrow frequency content is generally expected to be similar across the regions. Consequently, the natural frequencies are modeled as stochastic with equal means.

### Frequency depression, and in- and anti-phase synchronization

Accounting for the network dynamics is a more complex approach that is more realistic, especially when the overall coherence is not insignificant. The brain network model predicts that the distribution of the phase lags will always have a peak at 0, with an additional peak at ±*π* appearing for anti-phase synchronization.

Of crucial importance here is whether the frequency increases or decreases during synchronization. For lowest frequencies of electro-physiological brain signals, delays cause relative phases within the first quadrant, so the frequency is depressed regardless of the topology of the delays, whilst the phase locking is in-phase. Hence stronger nodes on average phase lag behind the weaker, for any arrangements of the natural frequencies. For higher frequencies, theoretical and numerical results show that for ordered networks both directions of the frequency shift are possible. However, in-silico brain dynamics exclusively shows frequency depression, so for equal stochastic frequencies better connected brain nodes phase-lag behind the weaker, whereas for anti-phase regime the *π* distance should be also accounted.

The frequency depression due to delayed interactions has wider importance than the 1:1 synchronization that is discussed here. Slowing down in an anatomically constrained dynamical system with noise has been shown to induce the whole-brain FC [64], by utilizing power to phase interactions. The latter are one of the mechanisms for cross-frequency coupling [65], which besides the well-known beta-theta interactions in the hippocampus [66], are also shown to occur for cortical signals [67].

The effects of signal mixing and spread due to volume conduction cause artificial synchrony between nearby sources that are alleviated with inverse source reconstruction techniques [68]. Nevertheless, linear mixing of signals from multiple sources can still lead to wrong coherence and phase synchrony estimates and this is commonly eliminated with interaction metrics that detect exclusively lagged interactions [28, 69]. This comes at the cost of an inability to detect true zero-phase lag interactions, which as we show, may be instead neurophysiologically meaningful and due to the coupling structure, as also suggested by other studies [33]. Including the actual zero-phase lagged interactions would henceforth potentially have an important impact on whole brain data analyses of M/EEG data, as it has been found that indices of Functional Connectivity sensitive to zero lag such as PLV tend to be more reliable within groups and across sessions [70, 71].

### Ordered versus complex networks

Although the general theoretical findings for the ordered networks still hold for the simulated dynamics over the connectome, the main contributor to their disparity is the complex spatio-temporal structure of the connectome. This is firstly reflected in the distribution of time-delays, which is neither exactly bi-modal *δ*, nor fully structured or random. Secondly, the weights of the links are not homogeneous for the nodes, as assumed by our approximation, but differ by several orders of magnitude. For the latter, combining other network measures, such as centrality or clustering coefficient [72], could potentially increase the predictability of the analysis. The remaining open issues of our brain network model are conceptual and are a common concern for most of the studies based on the connectomics. These are questions about the meaning of the weights and utilization of links, but also about the actual propagation velocity along tracts, which is shown to depend on large number of quantities [73].

### Methodological limitations

The level of significant coherence does not impact the overall architecture of phase lags, although when it is lower it mostly increases the variance of the results by flattening the distribution. However, the stricter level of significance can fail to capture phase-locking, especially between the hemispheres where the coherence is lower due to reduced wiring.

Increased variance of the phases can also indicate an alteration between different stable states. This often causes intermittent in- and anti- phase synchronization, when several peaks appear in the distribution of pair-wise phase lags. We showed that averaging of these non-stationary dynamics leads to improper description of phase relationships and can be avoided by differentiating of the separate regimes during the analysis. However, identification of time-dependent dynamics is a major challenge in analysis of biological signals [74].

Inherent variability of the frequencies or coupling strengths [48, 75] is another source of non-stationarity for which we demonstrated that the observed phase-lags depend on the overall statistics of the averaged parameters. Nevertheless, dividing the time-series to different epochs for more precise identification of phase lags for different regimes is also possible in cases when the non-autonomous forcing can be recovered [74], as well as quantification of the non-autonomicity [76].

Besides the notion of synchronization, functional brain connectivity can be also described by directed information flows [77], or effective connectivity [78, 79]. Bayesian frameworks [80, 81], although limited to instantaneous interactions, offer another approach for studying the connectivity between neural systems, by inferring coupling functions [82] that are spatially and frequency specific [42, 43].

## Supporting information

S1 Files Data and scripts. The folder contains the data used in this work together with the Matlab codes (scripts and functions) necessary to perform the simulations and the analysis. Data and codes are also available at https://gitlab.thevirtualbrain.org/spase.petkoski/plos_PLSNS.
(ZIP)
